# Long-Term Treatment with Aqueous Garlic and/or Tomato Suspensions Decreases Ehrlich Ascites Tumors

**DOI:** 10.1155/2014/381649

**Published:** 2014-06-30

**Authors:** Jenifer Bom, Patrícia Gunutzmann, Elizabeth C. Pérez Hurtado, Jussara M. R. Maragno-Correa, Silvia Regina Kleeb, Maria Anete Lallo

**Affiliations:** ^1^Environmental and Experimental Pathology Post-Graduation, Paulista University (UNIP), Rua José Maria Whitaker 290, 05622-001 São Paulo, SP, Brazil; ^2^Pharmacology Department, Federal University of São Paulo (UNIFESP), Rua Sena Madureira, 04021-001 São Paulo, SP, Brazil; ^3^Veterinary Medicine, University Metodista of São Paulo, Rua Alfeu Tavares 149, 09641-000 São Bernardo do Campo, SP, Brazil

## Abstract

We evaluated the preventive and therapeutic effects of aqueous suspensions of garlic, tomato, and garlic + tomato in the development of experimental Ehrlich tumors in mice. The aqueous suspensions (2%) were administered over a short term for 30 days before tumor inoculation and 12 days afterward, and suspensions at 6% were administered for 180 days before inoculation and for 12 days afterward. The volume, number, and characteristics of the tumor cells and AgNOR counts were determined to compare the different treatments. Aqueous 6% suspensions of garlic, tomato, and garlic + tomato given over the long term significantly reduced tumor growth but when given over the short term, they did not alter tumor growth.

## 1. Introduction

Cancer is a disease of complex etiology. It is now recognized that a great majority of human cancers, perhaps as many as 80–90%, are attributable to environmental factors [1]. A number of these factors, such as the association between smoking and lung cancer, a high-fat diet with breast and colon cancer, and several other malignancies, have been identified [2, 3]. The transformation of normal cells to cancerous involves three distinct phases: initiation, promotion, and progression [4]. Dietary habits are known to modify each of these phases [5]. Plants contain an extensive variety of compounds, some of which are strong modifiers of chemical carcinogenesis [6]. The prevention of cancer through the ingestion of vegetables and fruits has been suggested in human epidemiologic studies [1, 6]. The induction of apoptosis is currently recognized as a useful strategy to treat and prevent cancer, and a large number of natural dietary constituents have been reported to induce apoptosis in malignant cells [7, 8]. These findings are consistent with the observation that high consumption of fruits and vegetables is associated with reduced risk of various cancers; in particular, tomato and garlic are recognized to possess a wide range of beneficial effects [1, 9].

Garlic (bulb of* Allium sativum*), a common plant used as a food item as well as a medicinal herb in many countries of the world, is one of the most ancient plants reputed to have cancerostatic effects, as well as antiviral, antifungal, and antibacterial activities, and the ability to lower blood lipid levels and blood pressure [1, 10]. Garlic contains at least thirty-three sulfur compounds, several enzymes, and seventeen amino acids. Additional constituents of intact garlic include steroidal glycosides and lectins [1, 6]. The sulfur compounds are responsible for garlic's pungent odor and many of its medicinal effects [11]. The anticarcinogenic properties of garlic have been indicated in several studies [1, 12–14].

Beneficial effects of tomato (*Lycopersicon esculentum*) were observed against cancer of the pancreas, colon, rectum, esophagus, and breast [13, 15, 16]. Lycopene, which is found in tomato-based products, belongs to the carotenoid family, a group of more than 1000 plant and animal pigments involved in photosynthesis and photoprotection. Lycopene may be important in preventing prostate cancer [17, 18].

Transplantable experimental tumors have been used in studies of physical, chemical, viral, and hormonal carcinogeneses. Ehrlich tumor is a transplantable neoplasm from a malign epithelium, such as mammary adenocarcinoma in female mice. When inoculated intraperitoneally, the tumor grows in an ascitic form [19]. In this study, we compared the effects of aqueous garlic and/or tomato suspensions administered over a short and long term on the prevention and treatment of experimental Ehrlich ascites tumors in mice.

## 2. Material and Methods

### 2.1. Bioactive Compounds

Garlic bulbs and tomato paste (28° Brix, with 30.5 mg/100 g of lycopene) were bought from a local market (São Paulo City, SP, Brazil). The dehusked cloves of garlic were ground in a mortar and pestle, and the formed paste was diluted with distilled water to make 2% and 6% aqueous suspensions (w/v). The suspensions of garlic were prepared fresh daily before oral administration [16]. To prepare the tomato aqueous suspension, we mixed tomato paste and distilled water to obtain 2% and 6% suspensions (w/v). Finally, 2% and 6% aqueous suspensions of the two components (garlic + tomato) were made by diluting garlic and tomato paste in 200 mL of distilled water.

### 2.2. Ehrlich Tumor

The Ehrlich ascites tumor was maintained in 3-month-old female BALB/c mice in ascitic form under a week passage. In the experiment, the animals were intraperitoneally (IP) injected with 5 × 10^7^ viable tumor cells suspended in 0.3 mL of phosphate-buffered saline (PBS) or the same volume of PBS (vehicle solution). The suspension containing the tumor cells was prepared according to a previous study [19, 20]. The ascitic fluid was collected by IP puncture using a sterile insulin syringe. Ascites tumor cell counts were performed using a Neubauer hemocytometer, and the cells were found to be more than 99% viable by the trypan blue dye exclusion method [20].

### 2.3. Animals

Adult female inbred BALB/c mice (8 weeks) weighing 25.9 ± 2.4 g were housed in groups of five in a ventilated caging system in a temperature- and humidity-controlled facility with a 12 h light/dark cycle. The mice were fed a balanced, commercial diet ad libitum. Experiments were carried out in compliance with the principles of International Laboratory Animal Care and the European Communities Council Directive (86/809/EEC) and were approved by the local ethical committee.

### 2.4. Experimental Design

**Figure 1:**
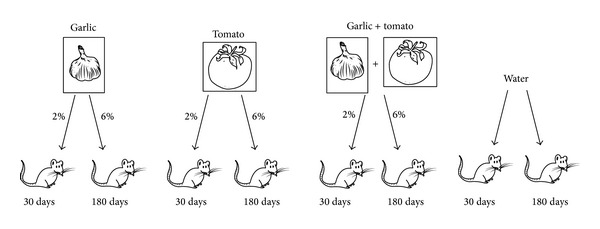
Experimental design. Eight groups were constituted, that is, 2% or 6% garlic and/or tomato and an untreated group that received only water. Animals receiving a 2% suspension were treated for 30 days, and those that received a 6% suspension were treated for 180 days.

The animals were divided into eight groups (*n* = 10 mice for group) to carry out two independent experiments, the first with short term administration of the compounds for 30 days and the second with the long term administration of aqueous suspensions for 180 days before tumor inoculation. The groups were formed according to the type of treatment (Figure 1): garlic: received aqueous garlic suspension (at 2% for 30 days or 6% for 180 days); tomato: received aqueous tomato suspension (at 2% for 30 days or 6% for 180 days); garlic + tomato: received aqueous garlic and tomato suspension (at 2% for 30 days or 6% for 180 days); control: received only water. The bioactive compounds were prepared as described above and offered ad libitum as the only source of water for the animals allocated in the garlic, tomato, and garlic + tomato groups. The exchange of these compounds was performed three times a week, and intake of the bioactive compounds as well as water consumption (control group) was measured and recorded for later analysis. All animals were weighed once a week to evaluate weight gain.

In the first experiment, the animals that received the 2% aqueous suspension and the control group were inoculated with experimental ascites Ehrlich's carcinoma cells (0.3 mL of 5 × 10^7^ cells, IP) on the 30th day, and they continued to receive the bioactive compounds. On the 12th day after tumor implantation, the animals were anesthetized, and the ascites fluid was collected for volume and cell number quantification. In the second experiment, after 180 days of treatment, 3 groups that had received long term treatment with aqueous suspensions and the control group were inoculated with Ehrlich tumors as described for the short term treatment groups.

### 2.5. Quantification and Qualification of Ehrlich Ascitic Tumor Growth

For Ehrlich tumor growth evaluation, the ascitic fluid present in the experimental and control mice was collected, the volume was measured, and the number of tumor cells was counted in a Neubauer chamber using the trypan blue dye exclusion method. The ascites fluid was centrifuged for 10 minutes at 200 g, the supernatant was discarded, and the solid volume was measured.

Smears from the cell suspension obtained from each animal were carried out and then submitted to a panoptic stain to determine the cell characteristics, dark cells/clear cells ratio, nucleus/cytoplasm ratio, and mean diameter of neoplasm cell nuclei using an immersion objective (1000x magnification). The mean nucleus diameter was only evaluated in mononucleated neoplasm cells.

The nucleolus organizer region was stained with silver (AgNORs), in accordance with a technique described by Ploton et al. [21] and modified by Aubele et al. [22]. Incubation was carried out in a humid and dark chamber at 40°C for 15 minutes. Characterizing and counting the number of AgNORs were carried out using an immersion objective. The number of cells and nuclei (to determine the nucleus/cytoplasm ratio and the number of NORs) was determined by studying the mean instability variation of the sample from which they originated (1000x magnification). The standard deviation and variation coefficient of the studied variables were approximately thirty cells/slide/animal.

### 2.6. Toxicity Study

Liver, kidney, lung, spleen, heart, and intestine nodes were collected, and representative slices were routinely processed for embedding in paraffin. Sections (5-*μ*m) were stained in H&E and examined for possible histopathological changes.

### 2.7. Statistical Analysis

The differences in mean values among different groups were tested and expressed as means ± SD. Statistical analysis was carried out using SPSS version 15.0 (Statistical Package for the Social Sciences, Chicago, IL). Data were expressed as means ± SD, and values of *P* < 0.05 were considered statistically significant. For the animals' body weights, the differences among groups were verified by ANOVA, while differences of other variables were investigated using ANOVA or the Kruskal-Wallis test (when the data were not normally distributed).

## 3. Results

In the first experiment, when treating the animals for a short period (30 days before inoculation and 12 days after inoculation), we observed that tumor growth was the same in all experimental groups, with no statistically significant differences between animals treated with aqueous suspensions of garlic, tomato, or garlic + tomato relative to the control. We observed the mean volume of ascites tumors, solid tumor mass, number of cells per mL, and the total number of tumor cells but found no significant differences between the groups (Table 1).

However, in the second experiment, we observed that the groups administered aqueous suspensions of garlic and garlic + tomato at 6% over the long term (180 days before tumor inoculation and 12 days after inoculation) showed significantly reduced tumor growth. The average volume of the tumor ascites fluid, the volume of solid mass, the tumor cell number per milliliter, and total tumor cells were significantly lower in animals receiving the aqueous suspension of garlic and garlic + tomato compared with the control group, which received only water (Table 1).

Regarding the group treated with the aqueous tomato suspension, although the average volume of fluid and of the solid mass tumor showed no significant difference when compared to the control group, the mean number of tumor cells per mL and total tumor cells were significantly lower than those of the control group.

In experiment 1, no statistically significant difference between the mean AgNOR dots obtained for the groups was observed, but, in experiment 2, groups treated with garlic, tomato, and garlic + tomato had significantly lower mean AgNOR dots (10.24 ± 1.45, 12.46 ± 1.87, and 11.37 ± 1.55, resp.) than the control group (19.22 ± 2.95) (Table 1).

The characteristics of the Ehrlich tumor cells differed among the groups. In the control group, a significantly smaller number of dark cells were observed, and cells that were clear and round with abundant, eosinophilic, vacuolated cytoplasm with few defined edges and large nuclei that were sometimes multinucleated were predominant. In the group treated with bioactive compounds, the opposite occurred; that is, a significantly smaller number of clear cells with predominance of dark cells were observed. Most cells were round, with moderate, basophilic cytoplasm that had a few vacuoles, defined edges, and hyperchromatic nuclei that varied from oval to round.

Neoplastic cells from the control group presented cytoplasmic and nuclear volumes larger than those of the groups treated with biocompounds. Morphometric analysis corroborated the differences in cell and nucleus size among the groups.

We observed that the garlic + tomato group had a lower body gain when compared to other groups, that is, garlic, tomato, and control, during the preinoculation period. However, after the experimental inoculation with Ehrlich tumors, there was no significant difference in weight gain between the groups (Table 2). The consumption of bioactive compounds did not significantly change relative to the water intake. No evidence of hepatic or renal toxicity was found by histopathological analysis.

## 4. Discussion

The beneficial anticarcinogenic and immunomodulatory effects of the agents in garlic [23], that is, water- and lipid-soluble allyl sulfides, can influence a number of molecular events involved in cancer, such as inhibiting mutagenesis, blocking carcinogen DNA adduct formation, scavenging free radicals, and blocking cell proliferation, differentiation, and angiogenesis. Although there is a large body of evidence supporting each of these and other mechanisms, there is a need for additional research to demonstrate whether these changes are causally related to cancer-preventive activity.

Carotenoids found in tomatoes are fat-soluble pigments responsible for the yellow, orange, and red colors in many fruits and vegetables [14]. Lycopene has the strongest antioxidant properties of the carotenoids, and higher intake has been associated with a reduced risk of many cancer types, including prostate, lung, and stomach [14, 15]. Recent work in cancer cell lines and animal models has demonstrated that lycopene can reduce the transcription of steroid-related genes, insulin-like growth factor-1 expression, and inflammatory signals, suggesting that these pathways may account, in part, for the inverse association between lycopene and the risk of some cancers [24, 25].

We observed that the bioactive compounds in garlic and/or tomato administered for a short period did not prevent or reduce the development of experimental Ehrlich ascites tumors in BALB/c mice. However, when we increased the concentration of the aqueous garlic and/or tomato suspension and administered the solution for a long period (for 6 months), the anticancer effects of garlic and/or tomato were observed because the growth of the tumor was lower in treated than in untreated animals.

The concentration of the compounds (2%) for the short term treatment was based on the results of Sengupta et al. [16], who also orally administered (by gavage) one milliliter of aqueous garlic or tomato suspension at a concentration of 2% for 12 weeks (84 days) prior to inducing experimental colon tumors in rat. They observed that a mixture of garlic and tomato was more effective in preventing colon cancer in rats. In our experiment, the compounds were spontaneously ingested by mice, although a difference in intake of the product compared to water intake by the controls has not been demonstrated. This difference in administration, as cited by the authors, may be one reason for the lack of active preventive compounds.

Carcinogenesis involves several stages: initiation, promotion, and progression. Tumor cells originate from normal cells that undergo changes in DNA (genetic) or mechanisms that control gene expression (epigenetic factors) in one or more location involved in controlling cell division and differentiation. The results of Sengupta et al. [16] may also differ from ours because they most likely used a chemically induced carcinogenesis model with clear phases of carcinogenesis in which the bioactive compounds act, while we used a tumor model in which cancer cells are transplanted and only progress.

When we increased the concentration of the compounds and administered them for an extended time (long term), the results showed that the animals treated with garlic, tomato, or garlic + tomato exhibited less tumor growth, demonstrating the preventive effects of biocompounds when given spontaneously in drinking water.

Ehrlich tumors are rapidly growing carcinomas with aggressive behavior. They are able to grow in nearly all mouse strains, which suggests that the recognition and immune responses to these tumors are independent of the MHC [26]. This characteristic suggests that controlling an Ehrlich tumor is more related to innate immunity, especially the inflammatory response, than to T cell responses [27]. The Ehrlich ascites tumor implantation induces per se a local inflammatory reaction, with increasing vascular permeability, which results in the formation of an intense edema and progressive ascites fluid and cellular migration [28]. This effect is most likely related to angiogenesis and growth factors that are induced by inflammation and necessary for tumor development.

Following treatment with a 2.5% aqueous garlic suspension by gavage, significant inhibition of cell proliferation and induction of apoptosis, as well as suppression of cyclooxygenase-2 (COX-2) activity, was observed [16]. The superior chemopreventive effect of garlic on skin carcinogenesis has also been demonstrated. Garlic induced elevated levels of antioxidants, accompanied by decreased lipid peroxidation and incidences of papillomas. Garlic treatments also downregulated COX-2 expression, a cancer progression marker, and decreased p53 and caspase-3 expression [29]. Unlike other studies that showed a strong influence of the bioactive compounds in garlic and tomato on the induction of experimental tumors, such as their antioxidant activity, it was demonstrated in this experiment that the bioactive compounds in garlic and tomato inhibit the proliferation of preestablished tumor cells of an experimental model consisting of Ehrlich tumor-implanted tumor cells. These findings reinforce evidence that the anti-inflammatory actions of these substances modify the peritoneal microenvironment and reduce other factors related to the genesis and development of tumors, for example, reduction of neovascularization, as these compounds can inhibit the activity of COX-2.

Indeed, it has been reported that this carotenoid is able to inhibit edema and the inflammatory response in different animal models by quenching singlet oxygen (^1^O_2_) or inhibiting the generation of superoxide (O_2_^−^) and nitric oxide (NO) from inflammatory leukocytes. Additionally, *β*-carotene possibly inhibits prostaglandin production by negative modulation of arachidonic acid oxidation via the cyclooxygenase pathway [16, 30]. Data show that Ehrlich tumor cells trigger an increase in prostaglandin E2 levels, which is detectable upon peritoneal washing of mice 24 hours after tumor implantation and remains elevated on days 6 and 10 of tumor development [28]. Prostaglandins have many biological effects in acute inflammation. Prostaglandin E2 is an important mediator of inflammatory vasodilatation and markedly potentiates the permeability and chemotactic effects of other mediators. In fact, increased levels of prostaglandins, most notably E2, have been detected in many malignant tumors. *β*-Carotene differentially modulated intercellular communication in the gap junction dependent on the dose, and this treatment could be rather harmful at high doses [31].

In the same way, it is possible that garlic and onion exert their anticarcinogen actions indirectly by different mechanisms, such as through inhibition of lipoxygenase and cyclooxygenase activities (an anti-inflammatory effect) [12, 32].

In the current study, the number of NOR dots was significantly lower in mice treated with 6% aqueous suspensions of garlic, tomato, and garlic + tomato than that of the control group. The NORs are DNA segments that encode ribosomal RNA [21]. Some nuclear proteins are associated with NORs and are stained black with silver methods (AgNOR proteins or AgNORs) [22]. It has been reported that AgNORs are related to the rate of cell proliferative activity [33]. Therefore, the decrease of AgNOR dots in groups treated with bioactive compounds over a long term (garlic, tomatoes, and garlic + tomato) shows that these products reduce the proliferative activity of experimental tumors.

Cell pleomorphism and the presence of several nucleoli and binucleated cells are also important characteristics of a tumor malignity [34]. Although the polymorphism was present in all cells of all groups, a greater nucleus/cytoplasm ratio relative to the control group, which is another important characteristic of malignant neoplastic cells, was less frequently observed in tumor cells of mice treated with the bioactive compounds over a long term (experiment 2).

We conclude that the aqueous suspension of garlic and/or tomato at 6% administered orally for a long term decreased Ehrlich tumor growth in an experimental model.

## Figures and Tables

**Table 1:** Average parameters of the Ehrlich tumors in the experimental groups treated with aqueous suspensions of garlic, tomato, or garlic + tomato over the short and long term.

Groups	Ascitic volume (mL)	Solid mass tumor (mL)	Number of cells by mL (×10^8^)	Total concentration of tumor cells (cells ×10^8^)	Number of NORs/nuclei
Experiment 1					
Garlic 2%	10.0 ± 1.95	3.2 ± 0.39	2.94 ± 0.89	11.58 ± 4.75	16.45 ± 1.95
Tomato 2%	11 ± 1.79	4.0 ± 0.36	3.52 ± 0.24	10.94 ± 5.65	14.85 ± 2.52
Garlic + tomato 2%	10.5 ± 2.11	3.9 ± 0.89	3.25 ± 0.44	10.96 ± 3.89	15.48 ± 2.23
Control	12 ± 1.90	4.1 ± 0.56	4.38 ± 0.95	12.47 ± 5.10	18.67 ± 2.83
Experiment 2					
Garlic 6%	8.0 ± 1.15∗	1.5 ± 0.39∗	1.15 ± 0.25∗	3.49 ± 0.86∗	10.24 ± 1.45∗
Tomato 6%	10 ± 1.97	3.0 ± 0.26	1.97 ± 0.16∗	6.03 ± 0.73∗	12.46 ± 1.87∗
Garlic + tomato 6%	7.9 ± 2.31∗	1.0 ± 1.17∗	1.00 ± 0.11∗	2.9 ± 1.60∗	11.37 ± 1.55∗
Control	11 ± 2.43	4.2 ± 0.90	3.46 ± 1.50	13.8 ± 8.10	19.22 ± 2.95

Data are represented as the means ± SD. *P < 0.05 in comparison to the control group (one-way ANOVA and Tukey-Kramer post hoc test).

**Table 2:** Mean weight gain for animals from the two experiments before and after inoculation with an Ehrlich tumor.

Experimental groups	Mean weight gain before inoculation (g)	Mean weight gain after inoculation (g)
Garlic	5.00 ± 2.04	4.99 ± 2.1
Tomato	6.39 ± 1.66	4.11 ± 1.1
Garlic + tomato	3.54 ± 1.32∗	3.80 ± 1.4
Control	5.15 ± 0.92	4.52 ± 1.3

*Data are represented as the means ± SD. *P < 0.05 in comparison to the control group (one-way ANOVA and Tukey-Kramer post hoc test).
